# Multiple roles of cardiac macrophages in heart homeostasis and failure

**DOI:** 10.1007/s10741-021-10156-z

**Published:** 2021-08-13

**Authors:** Aneta Moskalik, Justyna Niderla-Bielińska, Anna Ratajska

**Affiliations:** 1 Postgraduate School of Molecular Medicine, Medical University of Warsaw, Warsaw, Poland; 2 Department of Histology and Embryology, Medical University of Warsaw, Warsaw, Poland; 3 Department of Pathology, Medical University of Warsaw, Warsaw, Poland

**Keywords:** Cardiac macrophages, Macrophage origin, Cardiac macrophage phenotype, Macrophage functions, Heart failure

## Abstract

Macrophages are essential components of the immune system and play a role in the normal functioning of the cardiovascular system. Depending on their origin and phenotype, cardiac macrophages perform various functions. In a steady-state, these cells play a beneficial role in maintaining cardiac homeostasis by defending the body from pathogens and eliminating apoptotic cells, participating in electrical conduction, vessel patrolling, and arterial tone regulation. However, macrophages also take part in adverse cardiac remodeling that could lead to the development and progression of heart failure (HF) in such HF comorbidities as hypertension, obesity, diabetes, and myocardial infarction. Nevertheless, studies on detailed mechanisms of cardiac macrophage function are still in progress, and could enable potential therapeutic applications of these cells. This review aims to present the latest reports on the origin, heterogeneity, and functions of cardiac macrophages in the healthy heart and in cardiovascular diseases leading to HF. The potential therapeutic use of macrophages is also briefly discussed.

## Introduction

Macrophages are important components of both the innate and adaptive immune response [1]. Studies on these cells date back to 1882 when Ilye Metchnikoff, a Nobel Prize laureate, based on his observations, discovered the phenomenon of phagocytosis [2, 3]. According to these findings and further research macrophages are considered to be phagocytic cells (the relevant differences between macrophages, phagocytes, monocytes, and T-cells are listed in Table 1). Moreover, as a part of the adaptive immune response, macrophages are able to present antigens to T lymphocytes (called T cells), which initiate and modulate immune cell responses, and exhibit cytotoxic activity, especially towards tumor cells [1]. Unlike monocytes, which typically circulate in the bloodstream, macrophages are present in almost every tissue, where they play diverse roles in tissue homeostasis via their involvement in modulating inflammatory reactions (by secreting various mediators and/or cell–cell contact), angiogenesis, lymphangiogenesis; shaping the lymphatic vessel lumen diameter; regulating fibrosis, wound healing, metabolism in obesity and insulin resistance; sensing tissue osmotic pressure; and many others [4]. Tissue macrophages also have diverse origins (yolk-sac, fetal liver, bone marrow) and phenotypes [5]. They are generally divided into M1 and M2 populations, defined by the expression of specific (membrane bound and/or cytoplasmic) markers and the release of a plethora of mediators, cytokines, and growth factors [6]. M1 macrophages are characterized by the expression of CD80, CD86, and CD16/32 and secretion of proinflammatory cytokines, whereas M2 macrophages are anti-inflammatory and overexpress arginase-1 (Arg-1), CD206 (mannose receptor), and interleukin-10 (IL-10) [6]. However, due to the fact that macrophages are very plastic cells, the line between M1 and M2 phenotypes often blurs, which is related to macrophage-specific functions and depends on the tissue microenvironment in which they are located [4]. Nowadays, it is possible to distinguish macrophages residing in various tissues, based on different gene expression profiles and protein levels [7–9].

**Table 1:** Major differences between monocytes, macrophages, phagocytes, and T-cells

Type of cells	Origin	Markers	Function/properties
Monocytes	Bone-marrow-derived cells of myeloid-lineage	Ly6C^hi^ and Ly6C^low^ (in mice) CD14^++^/CD16^± ^classical, CD14^++^/CD16^+^ intermediate, CD14^+^/CD16^++^ nonclassical (in human)	Circulate in blood (with a half-life of ~ 3 days)[157] and/or reside in subcapsular space of the spleen [136]; constitute ∼10% of peripheral leukocytes in humans and ∼4% in mice; differ from macrophages by lack of F4/80. CD11b, expression, and low expression levels of CD68 and MHC-II [158, 159]; capable to phagocytose, and have vessel patrolling function; circulating and spleen-residing monocytes have the same transcriptomic profile [136]; have the ability to be rapidly mobilized in large numbers to inflamed sites throughout the body; differentiate into macrophages and dendritic cells [160]
Macrophages	Yolk sac-derived erythro-myeloid precursors bypassing monocyte intermediate; hematopoietic cells of fetal liver, local hematopoietic foci in prenatal organism, from blood/spleen monocytes that penetrate tissues, and from local proliferation of tissue macrophages; seed tissues early in embryonic development and continue later prenatally and postnatally	Major populations - M1 (pro-inflammatory, classically activated): CD68^+^/CD206^−^, CD86^+^, iNOS, IL-6, TNF-α; M2 (anti-inflammatory, alternatively-activated): CD206^+^/CD163^+^, Arg1 [161, 162], differ in terms of transcriptomic and protein profiles related to given tissue/organ microenvironment, e.g., high expression of CD11a and EpCAM for lung macrophages, VCAM-1 and CD31 for spleen macrophages, CD93 and ICAM-2 for peritoneal macrophages [7–9, 163]	Mononuclear phagocytes capable to phagocytose various foreign microorganisms, particles, dead /apoptotic/senescent cells [164]; moreover having additional diverse functions: such as niche cells for erythropoiesis [165, 166], promoting/regulating of angiogenesis [167], lymphangiogenesis [168], vessel wall lumen regulation [169], wound healing, fibrosis, cell/organ involution during embryonic development (capillary regression in pupillary membrane) [170], surfactant excess removal in lung development [171], neuronal/synaptic pruning [172], osmotic/tissue volume/fluid sensing [106], insulin sensing in adipose tissue [173], norepinephrine metabolism [116], reverse cholesterol transport [174], iron recycling in spleen and liver [175]
cTM, cardiac tissue macrophages	As other macrophages; and from hemogenic endothelium of cardiac cushion tissue	As described in Table 2	As described in Table 2
Phagocytes	Bone marrow myeloid-lineage-derived precursors (granulocytes, monocytes, macrophages—professional phagocytes); epithelial cells, fibroblasts, dendritic cells—phagocytose with slower rate and less specifically—nonprofessional phagocytes [176]	Capable to recognize phagocytic receptors (“eat-me signals”)—phagocytosis is mediated by various pathways	Cells exhibiting phagocytic activity; phagocytosis is the uptake by the cell of relatively large particles (> 0.5 µm) into vacuoles, by mechanisms that are clathrin-independent and usually require actin polymerization. Particles involved are microorganisms (bacteria, viruses), dead cells, tissue remnants, tumor cells, senescent erythrocytes, ejected erythroblastic nuclei, spermatogonia in spermatogenesis, etc. [47, 177]
T-cells	Bone marrow hematopoietic common lymphocyte progenitor; differentiate in thymus gaining T cell receptor (TCR)	T-helper (CD4^+^), T-cytotoxic (CD8^+^), T-regulatory (T-reg; CD4^+^CD25^+^Foxp3^+^)	Seed various tissues, including cardiac; T-cells comprise about 3% of total cardiac leukocytes [178]. CD4 lymphocytes mediate inflammation and age-related cardiac dysfunction, as lack of these cells reduce inflammatory cytokine production [179]. Aged hearts have more CD8 than CD4 lymphocytes [179]. T-reg cells exhibit beneficial cardioprotective effect after myocardial infarction [180]

Cardiac tissue macrophages (cTMs) are immune cells located in myocardium, where they perform many functions, both in maintaining physiological tissue homeostasis and in many pathological processes. However, the mechanisms of these functions are still not fully elucidated. This review reports on the origin, heterogeneity, and functions of cTMs in healthy myocardium and in cardiovascular diseases leading to heart failure. In addition, we discuss possible future therapeutic applications with the use of (modified) macrophages.

## Cardiac macrophage origins

In the past, macrophages were thought to develop only from blood monocytes [10]. However, this theory was later challenged by many authors [11–13], indicating that adult heart macrophages originate from various other sources and are able to proliferate in situ, augmenting the resident macrophage population. In addition, the origin of macrophages is believed to be related to their specific functions. Therefore, to better understand this origin-function relationship, let us take a closer look at macrophage ontogenesis.

During embryonic development, the first tissue macrophage precursors come from the yolk sac and then from fetal liver mononuclear cells, which colonize the heart and contribute to the formation of resident cardiac macrophages [14]. In the yolk sac, there are two waves of erythro-myeloid progenitors (EMPs). The first wave, beginning from embryonic day (E) 7.25 in mice, produces locally primitive erythroblasts, megakaryocytes, and macrophages without monocytic intermediates. Subsequently, at E8.25, the second wave-derived EMPs migrate into the fetal liver, where they give rise to definitive hematopoiesis, differentiating into all hematopoietic cell lineages, including monocytes [15–18]. First- and second-wave-derived EMPs could be distinguished by the lack of or dependence on c-Myb, respectively [16, 19, 20]. Primitive yolk sac macrophages migrate to the embryonic heart about E9.5, and these macrophages can be easily detected in the heart between E10.5 and E12.5 [21, 22]. The second wave, monocyte-like macrophages, which derive from the fetal liver, appear between E12.5 and E17.5. From E17.5 to adulthood, bone marrow-derived mature macrophages seed all tissues, including the heart [13, 14, 16]. Moreover, a recent report indicates hemogenic endocardium of the endocardial cushions as an additional source of cTMs. These cTMs, which emerge in developing mice at E9.5, exhibit an intensive phagocytic activity, then proliferate in situ, seeding the cushion tissue, and are indispensable for development and remodeling of the cushion tissue and, subsequently, valves [23].

Yolk sac macrophages are characterized as CD45^+^F4/80^hi^CD11b^lo^MHC-II^lo^CX3CR1^hi^, while the cells derived from fetal liver monocytes are CD45^+^F4/80^lo^CD11b^hi^CX3CR1^lo^ [13]. Furthermore, the expression of the CCR2 marker also facilitates the distinction of macrophages by their origin. In the developing heart, CCR2^−^ macrophages derive from primitive yolk sac progenitors and colonize the subepicardial space and the subepicardial part of the myocardial wall [13, 24], whereas fetal monocyte-derived macrophages express CCR2^+^ and occupy the endocardial trabeculae [25]. After birth, most cardiac macrophages of embryonic origin have a high capacity for self-renewal via in situ proliferation during homeostasis [26, 27]. However, with aging, cTM ability to proliferate gradually declines and the cells are increasingly replaced with monocyte-derived macrophages [13, 26–28]. In a healthy adult heart, all CCR2^+^ macrophages have been fully replaced by blood monocytes, while 70% of CCR2^−^ macrophages have self-renewed and 30% of them have been replaced by monocytes [13, 26, 29]. Interestingly, other tissue macrophages, e.g., alveolar macrophages, microglia, and Kupffer cells, are able to proliferate in situ in a steady-state, without becoming replaced by monocytes [30–32], whereas intestinal macrophages are being continuously replaced by blood-derived monocytes over the entire life span [33].

In conclusion, the adult heart consists of mixed populations of cardiac tissue macrophages in terms of their origin. The proportions of macrophages of various origins change with aging and with progression of various cardiovascular diseases, depending on the sex and on the type of cardiovascular dysfunction. Thus, macrophages may derive from embryonic precursors, blood monocytes, or hemogenic endocardium and increase in number by in situ proliferation. When homeostasis is disrupted, for example, after myocardial infarction (MI) that is associated with inflammation, blood monocytes are recruited to the heart and give rise to a new pool of macrophages [27].

## Cardiac tissue macrophage phenotypes

Early studies on mouse cardiac tissue macrophages characterized them as cells bearing the CD45^+^CD11b^+^CX3CR1^+^ markers [34]. Moreover, macrophages express other distinctive markers such as F4/80 and CD14, gaining CD64 and tyrosine-protein kinase Mer (MerTK) during heart development. Expressing of CD206 (Mrc1) and CD163 markers makes macrophages similar to alternatively activated M2 macrophages in a steady-state in adult mice. Mouse cTMs express negligible levels of Arg1 which is considered as a typical M2 marker [34]. There are many attempts to define cTM phenotypes and assess their subpopulations; most of these attempts are based on having selected antibodies bind with specific macrophage markers. Mouse cTMs can be characterized by various combinations of the absence and/or presence of CX3CR1 and MHC-II; and based on the expression of these markers, cTMs can be divided into four populations. The most numerous population and one with the most proliferative capacity is the CX3CR1^+^MHC-II^−^ population, which with aging, is gradually replaced by CX3CR1^−^ and MHC-II^+^ cells [26]. CX3CR1^+^ cells were previously reported to diminish in number with age [35]. The CX3CR1 molecule is involved in monocyte crawling or “patrolling” a vessel lumen, monocyte diapedesis across blood vessel walls, cell migration, chemotaxis, and angiogenesis, as well as apoptosis inhibition by upregulating Bcl2 required for cell survival. It is also involved in modulating inflammatory myeloid cell recruitment [36–38] (macrophage markers in relation to their functions are listed in Table 2). Ablation of CX3CR1^+^ macrophages increases mortality and augments peri-infarct fibrosis after myocardial infarction in mice [28]. Epelman et al., who conducted a detailed flow cytometric analysis of cardiac macrophages, showed that cTMs are represented by various populations: the main Ly6C^−^MHC-II^hi^CX3CR1^hi^CD206^int^ population, containing a smaller subset expressing CD11c^hi^, and a population expressing Ly6C^−^MHC-II^lo^CX3CR1^int^CD206^hi^CD11c^lo^. Ly6C^+^MHC-II^±^CD206^+^ cells constituted about 2% of all cTMs; moreover, a population of monocytes with a lack of MerTK and CD206 was observed [13]. Recent studies using fate mapping and single-cell transcriptomics define cTMs based on Lyve-1, MHC-II, and T-cell immunoglobulins and mucin domain-containing 4 (TIMD4) expression, the latter being involved in the clearance of apoptotic cells (efferocytosis) [28, 39, 40]. Accordingly, in a steady-state, the adult mouse heart consists of four cTM populations: TIMD4^+^LYVE1^+^MHC-II^lo^CCR2^−^ (self-renewable), TIMD4^−^LYVE1^−^MHC-II^hi^CCR2^−^ (partly replaced by monocytes), and two CCR2^+^MHC-II^hi^ groups, differing by their levels of expression of various genes, such as Irf7, Ifit1, and Isg20 (entirely substituted by monocytes) [28]. CCR2^−^ macrophages play an important role during fetal coronary vessel development and maturation, being responsible for the selective expansion of perfused vasculature, and mediation of primitive coronary plexus remodeling [25]. In the adult heart, CCR2^−^ are responsible for orchestrating tissue repair [41]. CCR2^+^ macrophages do not have any specific function defined during heart development; however, in the adult heart with ischemia–reperfusion injury, these macrophages induce alpha-smooth muscle actin (αSMA), lysyl oxidase, and collagen type 1 alpha 2 (Col1a2) expression in cultured fibroblasts, thus exhibiting profibrotic activity [42]. Additionally, cTMs support capillary homeostasis in adult myocardium and express proangiogenic factors, e.g., insulin-like growth factor 1 (IGF-1) and resistin-like molecule α (Rentla/Fizz-1) [43].

**Table 2:** Selected populations of cardiac macrophages and their functions

Main function	Population of cardiac macrophages	Characteristic
Phagocytosis	MHC-II^lo^CCR2^−^	Apoptotic and necrotic cells clearance The highest level of MerTK [50]
Electrical conduction	a) MHC-II^hi^CCR2^hi^ b) MHC-II^hi^CCR2^lo^ c) MHC-II^lo^CCR2^lo^	a,b) Generation of transient electrical signals, propagation of electrical signal by Cx43-gap junction coupling with cardiomyocytes b,c) Controlling heart rhythm and the heart beat owing to expression of ion channels [56]
Vessel patrolling	Monocytes: CX3CR1^hi^Ly6C^−^ (mouse) CX3CR1^hi^CD14^dim^CD16^+^ (human)	Recognition and removal of damaged luminal cells; scavenging micrometric particles at steady-state [37, 38, 62]
Arterial tone regulation	Lyve-1^+^	Regulation of vascular tone and collagen production by mural cells [63]
Osmoregulation	TonEBP^+^	Regulation of blood pressure, extracellular fluid, and solute volume Acting via TonEBP/VEGF-C signaling pathway Mediating lymphangiogenesis [105, 106]
Proinflammatory	CCR2^+^MHC-II^hi^ (mouse) CCR2^+^HLA-DR^hi^ (human) Ly6C^hi^ CCR2^+^	Monocyte-derived Promote inflammatory response [41, 137, 138]; produce IL-1b, increase cardiocyte necrosis, replacement fibrosis, worsen systolic activity in acute inflammation [76]; systemic inflammation triggers CCR2^+^ cardiac macrophage invasion and promote interstitial collagen deposition what stiffens myocardial wall [76, 181] resulting in diastolic dysfunction
Anti-inflammatory	CCR2^−^MHC-II^lo/hi^(mouse) CCR2^−^HLA-DR^hi^ (human) Ly6C^lo^ CCR2^−^	Embryonic origin Proangiogenic function Fetal coronary vessel development and maturation Reparative functions [25, 41] Wound healing Reduction of inflammation (IL-10 production) after acute phase, preservation of cardiac function; promotion of collagen deposition, fibrosis, and angiogenesis [142]
Heart valve remodeling	a) CD301b^+^ b) CD206^+^	a) Inflammation and fibrosis within the valve [148] b) Tissue repair promotion and regeneration [45]

A human adult heart contains similar populations of macrophages as the heart of a mouse. These cells could also be divided into CCR2^−^ tissue-resident macrophages, which renew through local cell proliferation, and CCR2^+^ cells, which derive from blood monocytes and have the ability to proliferate. Human and mouse cTMs can be distinguished based on the fact that human CCR2^−^ populations are mostly HLA-DR^hi^, while mouse CCR^−^ populations are MHC-II^lo^ and MHC-II^hi^. However, no functional differences have been demonstrated so far between CCR^−^MHC-II^lo^ and CCR^−^MHC-II^hi^. Human CCR2^−^ macrophages have reparative functions as well, and they express extracellular matrix components, such as SLIT3, and growth factors, such as IGF-1 and platelet-derived growth factor C (PDGF-C), whereas CCR2^+^ macrophages are associated with initiating inflammation, are characterized by the expression of several other receptors, and secrete chemokines, and inflammatory mediators, for instance interleukin-1β (IL-1β), and interleukin-6 (IL-6). Moreover, inflammation-associated macrophages were shown to participate in left ventricular systolic dysfunction and express genes, such as matrix metalloprotease-9 (MMP-9) and a metallopeptidase inhibitor 1 (TIMP1), involved in detrimental cardiac remodeling [41, 44].

Macrophages were also detected in heart valves, both in humans and in mice. Hulin et al. demonstrated the presence of various leukocyte populations in heart valves, especially in the regions of enhanced biomechanical stress (valve leaflet commissures and distal tips) [45]. Most of these leukocytes are F4/80^+^ macrophages, additionally expressing CD206 and/or MHC-II markers, and dendritic cells. Moreover, during postnatal valve development and remodeling, the number of these macrophages increases [45].

## The role of macrophages in the healthy cardiovascular system

### Protection: phagocytosis and immune defense

As phagocytosis is the key function of macrophages, these cells play a major role in defending the body against pathogens and eliminating apoptotic cells in a steady-state [46, 47]. After experimental infection of myocardium with parasites, the density of cardiac macrophages increases; macrophages assume stellate shapes and gain the expression of CD206, RELMα, and YM1, markers of the M2 phenotype [48]. After infection with bacteria, resident macrophages also exhibit the ability to engulf microorganisms within a short time [27]. In addition, macrophage receptors can recognize phosphatidylserine (PtdSer) on apoptotic cell surfaces either directly (e.g., TIMD4) or indirectly (e.g., MerTK, which binds soluble “bridging” proteins, including Gas6/Pros1) [49]. One crucial regulator of activation and function of resident macrophages in healthy and injured hearts is MerTK. This receptor mediates efferocytosis and promotes anti-inflammatory response. The highest level of MerTK was reported in MHC-II^lo^CCR2^−^ population whereas the loss of this receptor reduces efferocytosis of apoptotic cardiomyocytes [50, 51]. Moreover, cTMs secrete molecules, such as complement component 1q (C1q), galectin-1 (LGALS1), growth and differentiation factor 15 (GDF15), resolvin E1 (RvE1), that facilitate apoptotic debris clearance, inhibit leukocyte infiltration, and consequently lead to immune quiescence and a maintenance of homeostasis [43, 52]. Other genes related to dampening local inflammation that are expressed by cTMs are the genes for IL-10, IGF-1, and V-set and immunoglobulin domain containing 4 (Vsig4) [34]. The macrophage activity to engulf apoptotic bodies has been also detected during normal heart development and is crucial to eliminate excess tissue, i.e., by shortening of the outflow tract and endocardial cushion remodeling [53, 54]. Endocardium-derived macrophages, due to their significant phagocytic ability, are essential for valve formation during embryogenesis [23].

Recent studies have shown that macrophages maintain cardiac myocyte mitochondrial homeostasis during normal energetic turnover by eliminating dysfunctional mitochondria and other impaired organelles. As defective mitochondria are released from cardiomyocytes in subcellular particles (exospheres), macrophages easily engulf them with the aid of MerTK receptors. Macrophage or MerTK ablation contributes to metabolic dysfunction of the heart. This indicates that MerTK-mediated phagocytic activity of cTMs supports normal heart function and cardiac homeostasis [55].

### Electrical conduction

According to recent reports, resident macrophages located in the atrioventricular (AV) node directly modulate electrical properties of cardiac myocytes owing to macrophage coupling with cardiomyocytes via Cx43-containing gap junctions. This intercellular coupling leads to rhythmic macrophage depolarization and modulates the resting membrane potential and action potential of cardiomyocytes. Gene clustering of these macrophages reflects three macrophage subsets within the AV node: MHC-II^hi^CCR2^hi^, MHC-II^hi^CCR2^lo^, and MHC-II^lo^CCR2^lo^ [56].

The first two subpopulations share genes that are responsible for generating transient electrical signals (*Kcnj2*, and *Kcnq1*) and for propagating the electrical signal (*Scn2b*, whose alteration causes atrial fibrillation). Therefore, these macrophage populations are similar to AV node pacemakers. The other two macrophage subpopulations (MCHI-I^hi^CCR2^lo^ and MHC-II^lo^CCR2^lo^) share genes responsible for the sodium/calcium exchanger (*Slc8a1*) and heart rhythm control (*Ank2* = ankyrin2), therefore, are responsible for electrical signal propagation. The sinoatrial (SA) node also contains abundant macrophages. Both human and mouse SA and AV node macrophages are spindle-shaped with long processes to make contact with stromal cells. Cx43 has low expression in the compact zone of the AV node but a higher expression in the lower nodal bundle, which may reflect a different density of gap junction-associated macrophages propagating electrical signals in separate areas of the cardiac conduction tissue. Whether other immune cells present in this area are also coupled with cardiomyocytes via C43-containing gap junctions has not yet been elucidated [56, 57]. In addition, macrophage depletion triggers AV block, which demonstrates their indispensability to the process of electrical conduction in the AV node in a steady-state [56]. Moreover, macrophages may cause conduction abnormalities [56], such as atrial fibrillation, ischemia-induced ventricular arrhythmias or arrhythmias associated with various inflammatory reactions (e.g., Lyme disease, Chagas, viral myocarditis) [58–61].

### Vessel patrolling

A population of monocytes is involved in vessel patrolling during a steady-state and inflammation. This function is performed by crawling along healthy vessel endothelium with the involvement of lymphocyte function-associated antigen-1 (LFA-1) and CX3CR1 receptors, which also provides immune surveillance of surrounding tissues. The major function of a crawling monocyte is to recognize and remove damaged cells that arise in normal healthy tissues as a result of regular cell turnover. This mechanism is independent of the direction of blood flow and is slower than the typical rolling process. Patrolling monocytes, which are Ly6C^−^ in mice and CD14^dim^CD16^+^ in humans, have the ability to differentiate into tissue macrophages [37, 38, 62].

### Arterial tone regulation

The aorta is the main artery transporting oxygenated blood from the heart to the rest of the body. Maintaining appropriate aortic structure and function is very important, since aortic damage leads to cardiovascular diseases. In the steady-state, Lyve-1-expressing macrophages located perivascularly around the mouse aorta regulate vascular tone and collagen production by mural cells. In preventing collagen deposition, arterial fibrosis, and arterial stiffness, macrophages use a mechanism involving MMP-9 activation via Lyve-1-hyaluronan interaction [63].

## The role of macrophages in heart failure

Heart failure (HF) is an increasingly common world health problem in the human population, often with poor prognosis [64]. There are three types of heart failure: HF with preserved ejection fraction (HFpEF), defined by left ventricular ejection fraction (LVEF > 50%), HF with reduced ejection fraction (HFrEF): LVEF < 40%, and HF with mid-range ejection fraction (HFmrEF): LVEF of 40–49%, which differ in etiology and pathophysiology [65, 66]. These types differ in terms of their risk factors, comorbidities, course, the cellular profile of inflammation, the extent and time course of endothelial dysfunction, as well as in terms of nitric oxide (NO) decreased bioavailability, cardiac hypertrophy and myocardial cell stiffness (due to titin modifications, perivascular, interstitial and replacement fibrosis, different levels of collagen cross-linking, altered intercellular communications, e.g., cardiac calcium signaling, and other subcellular/molecular modifications), and response to treatment [67]. A network analysis demonstrated that the biomarkers of HFpEF and HFrEF are associated with different inflammatory profile responses and different extracellular matrix reorganization [68]. In HFpEF, extracardiac comorbidities promote systemic inflammation, coronary microvascular endothelial dysfunction, interstitial fibrosis, and impaired cardiomyocyte relaxation; these pathological processes culminate in LV remodeling and diastolic dysfunction. Common extracardiac risk factors for HFpEF include advanced age, female sex, renal dysfunction, pulmonary congestion, and metabolic syndrome-associated factors such as obesity, diabetes mellitus, and hypertension. Therefore, HFpEF is considered to be a multisystem disorder involving the heart, lungs, kidneys, skeletal muscle, adipose tissue, vascular system, and immune and inflammatory signaling [66]. Left ventricular (LV) remodeling in HFpEF with stiff LV (diastolic dysfunction) due to more intensive deposition of collagen I than collagen III is presented by collagens deposited in the interstitium and perivascularly. Moreover, there are increased serum levels of profibrotic cytokines: monocyte chemoattractant protein 1 (MCP-1), transforming growth factor β (TGF-β), and IL-6. Conversely, HFrEF is associated with cardiomyocyte cell death and consequences of inflammation, endothelial dysfunction, and replacement fibrosis, which lead to the development of systolic dysfunction. HFrEF is related to volume overload, myocardial infarction, myocarditis, valvular disease, and might be a result of genetic mutations [69, 70]. HFmrEF is an intermediate stage that could progresses to HFpEF or HFrEF and appears comparable to HFrEF, at least in its responsiveness to neurohormonal blocking agents [65, 67].

**Fig. 1:**
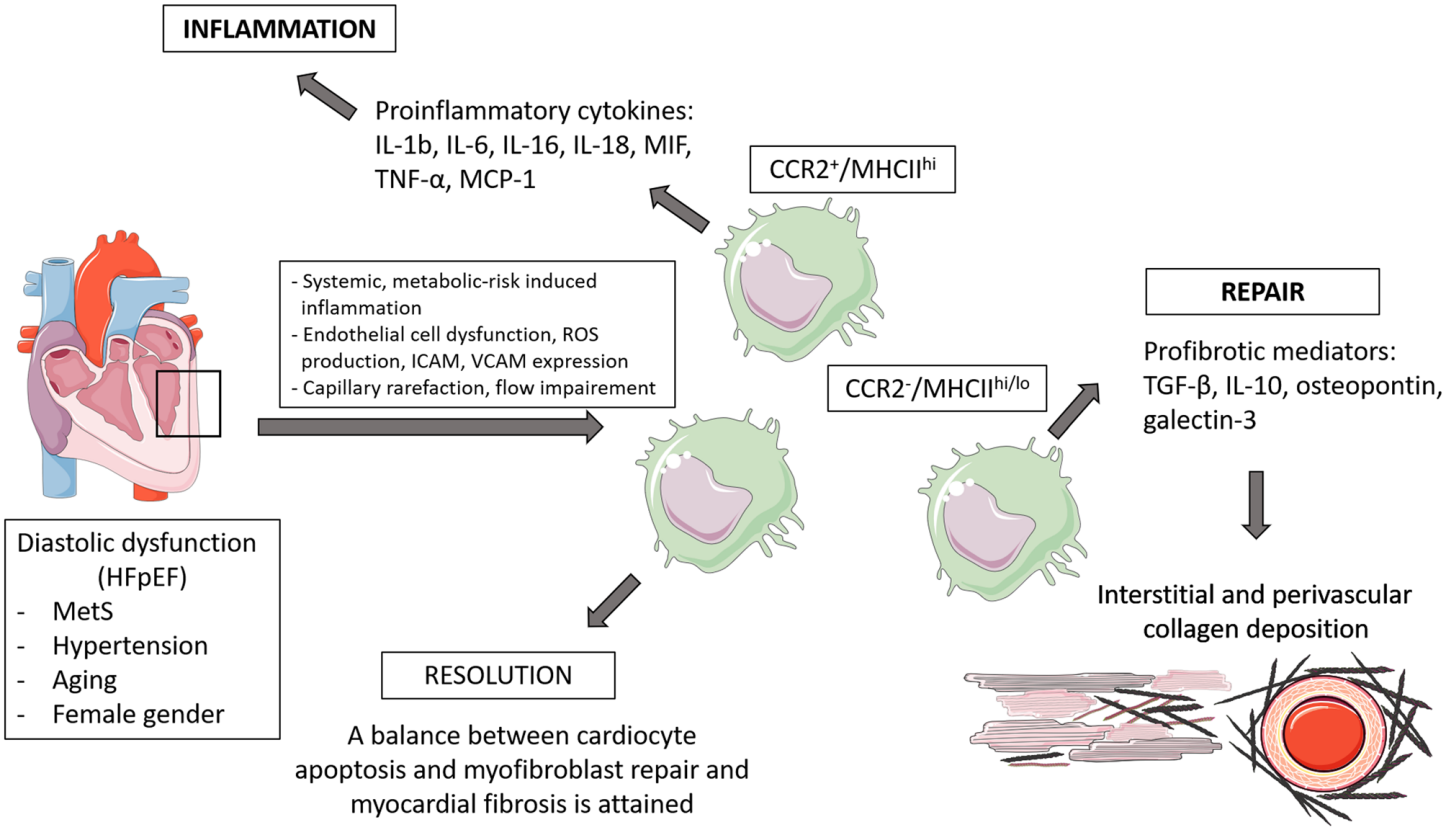
Schematic presentation of major macrophage-mediated cardiac remodeling pathways in development of HFpEF

**Fig. 2:**
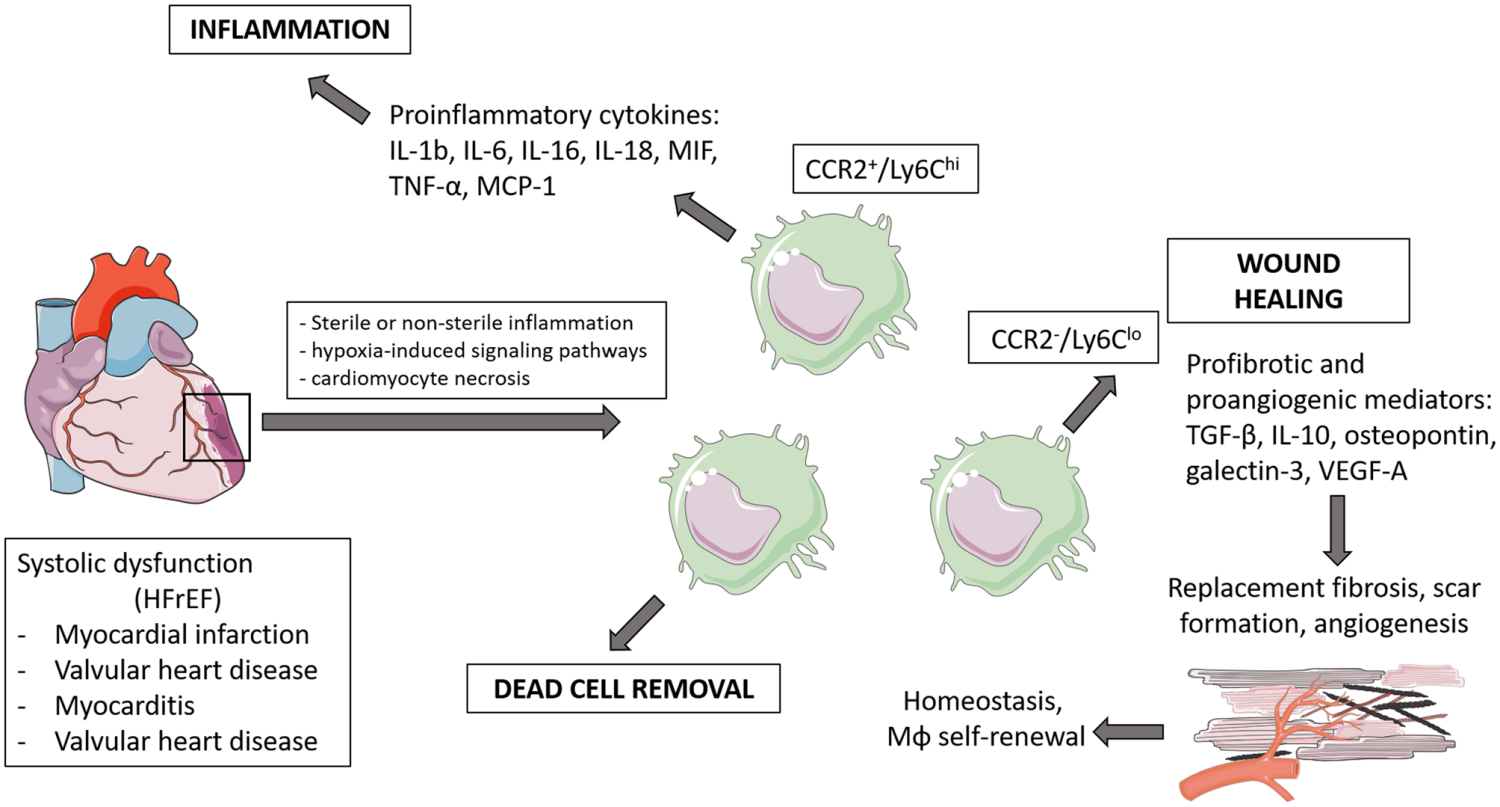
Schematic presentation of major macrophage-mediated cardiac remodeling pathways in development of HFrEF

Macrophages perform important functions in HF development and progression [71] (As presented on schematic Figs. 1 and 2, created using artwork from Servier Medical ART). However, detailed mechanisms are still not completely understood. Multiple pathways leading to HF seem to be triggered by macrophages, depending on the phenotypic character of these cells defined by their secretome, miRNome [72], and genetic (mRNA) profile. Nevertheless, it is known that the role of macrophages in hypertension, obesity, diabetes, renal dysfunction, which are risk factors leading to HF, is crucial [69, 70]. A thorough analysis of macrophage functions in cardiac pathologies may contribute to finding future therapeutic targets, which are particularly needed in HFpEF, since no pharmacological agents have meaningfully improved outcomes.

**Fig. 3:**
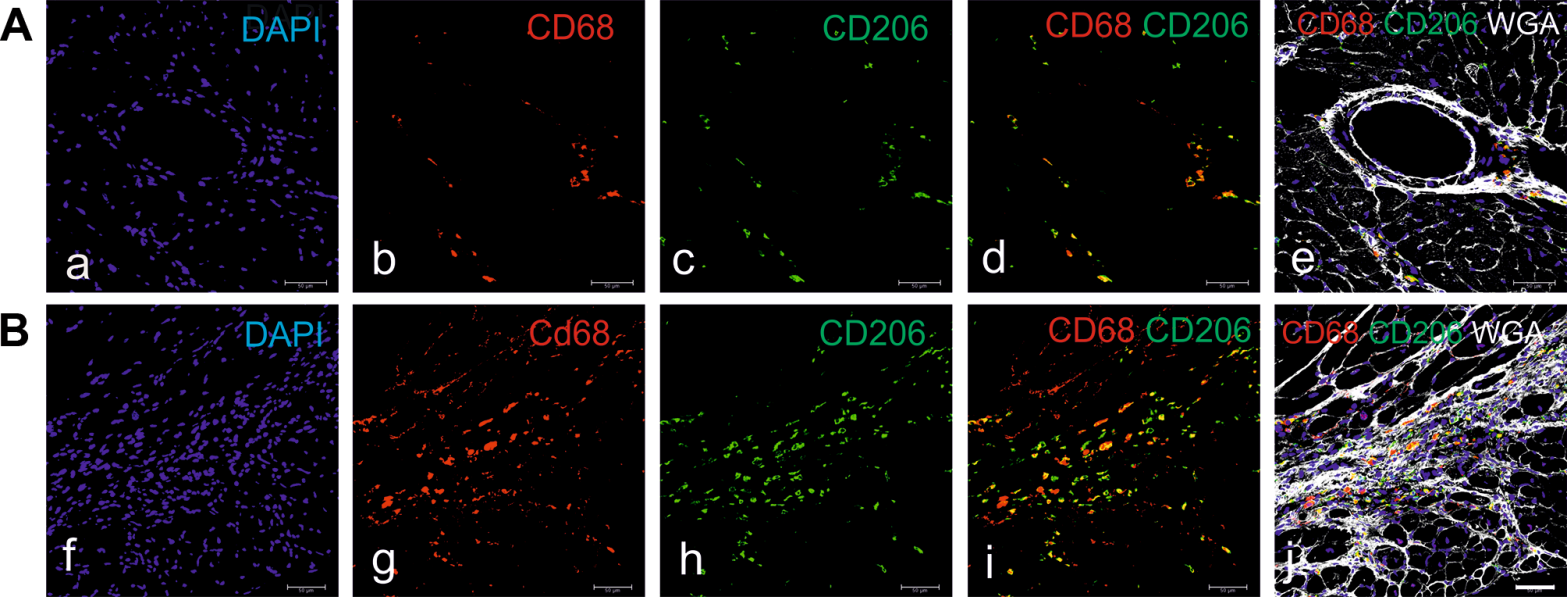
Perivascular (**A**) and scar-associated (**B**) macrophages in the myocardium of hypertensive db/db mice. Db/db mice were treated with angiotensin II for 4 weeks via infusion from subcutaneously implanted minipumps. The hearts were harvested at the age of 21 weeks, frozen, and immunostained as indicated on panels. Analysis was performed under a confocal microscope (Leica, Wetzlar, Germany). WGA, wheat germ agglutinin demarcates cell borders. Scale bar, 50 μm

Myocardial wall stiffness with diastolic dysfunction caused by interstitial fibrosis is a key characteristic of HFpEF. Profibrotic activities of macrophages have been described to proceed via various pathways. For example, macrophage-derived IL-10, which is secreted in excess during the development of diastolic dysfunction induced either by hypertension or aging, promotes profibrotic mechanisms, both in mice and humans. This process is initiated through an intensive recruitment of the circulating monocytes derived from bone marrow and spleen to the heart, which increases cardiac macrophage density [73]. Increased numbers of CD68^+^ macrophages and augmented fibrosis associated with inflammation were also confirmed in the aging rat model of pre-HFpEF, obese ZSF1 rats with HFpEF, and other animal models (our own observations) (Fig. 3) [74, 75]. Macrophages start to secrete IL-10, and their phenotype changes to profibrotic (characterized by the cell marker MHC-II^hi^). Although IL-10 production is considered beneficial, since it dampens inflammation and promotes tissue repair, in this case, it is excessive and harmful. Increased amounts of IL-10 contribute to the production of osteopontin, galectin-3, proteases, and MMPs, which in consequence induces myofibroblast activation and deposition of collagen. This increases myocardial stiffness and promotes diastolic dysfunction [73, 76]. It is worth noting that IL-10 could be also released by other immune cells, such as dendritic cell subsets, T-cell subpopulations, and B cells [77], by isolated cardiomyocytes stimulated by LPS [78], and by sham-operated hearts and in lesser amount by hearts after myocardial infarction [79].

HFpEF is strongly related to obesity and metabolic syndrome (MetS), which is accompanied by increased epicardial fat deposits and inflammation. After adipocyte death and independently, under the influence of hypoxia-inhibitory factor-1α (HIF-1α), M1-type macrophages are recruited. They release IL-1β, IL-6, tumor necrosis factor α (TNFα), and MCP-1, which may contribute to cardiac fibrosis and diastolic dysfunction. HIF-1α promotes also a profibrotic transcriptional program in myofibroblasts, involving collagen I, III, and IV deposition, lysyl oxidase, and elevated expression of TIMP1 [80]. In a mouse model of chronic angiotensin II-induced hypertension, cTMs also release galectin-3, a protein involved in myocardial fibrosis promotion [81]. Likewise, a profibrotic profile of macrophages was demonstrated in in vitro studies, when monocytes of healthy donors cultured with HFpEF patient serum differentiated into macrophages [82].

Interestingly, macrophages take part in perivascular fibrosis, which is more severe in HFpEF than in HFrEF [83, 84]. In a pressure overload model associated with LV diastolic dysfunction, macrophage recruitment in perivascular areas is mediated by increased level of MCP-1, and intercellular adhesion molecule 1 (ICAM-1) expression on intramyocardial artery endothelial cells. As a result, macrophages secrete TGF-β and other profibrotic cytokines, which activate fibroblast proliferation and subsequent fibrosis [84, 85]. Excessive perivascular fibrosis causes an impaired oxygen and nutrient supply due to a diminished blood flow, which leads to adverse cardiac remodeling [86]. Moreover, MCP-1 is upregulated in the serum of patients with HFpEF compared with the MCP-1 levels in the serum of healthy individuals [87], and TGF-β is an independent biomarker, which helps distinguish HFpEF from HFrEF [88]. Microvascular dysfunction also contributes to HFpEF. It leads to capillary rarefaction, thereby resulting in impaired blood flow and deficient tissue nutrient and oxygen supply. Macrophages stimulated with lipopolysaccharide (LPS), interferon-gamma (IFN-γ), or under tissue hypertensive conditions, activate Nf-ĸb, upregulating ICAM, MCP-1, and IL-6, thus causing endothelial cell (EC) dysfunction [89]. Recently, cardiac lymphatic vessel dysfunction and rarefaction have been described as a result of MetS. Imbalance in tissue fluid homeostasis and inflammatory cell trafficking would lead to cardiac edema, leading to fibrosis and diastolic dysfunction [90]. Moreover, considering that HFpEF is associated with systemic inflammation, microvascular ECs are activated, which prompts enhanced expression of adhesion molecules, e.g., vascular cell adhesion molecule (VCAM), ICAM-1, or E-selectin. Through upregulating NADPH oxidase 2 (NOX2) expression, macrophages promote high oxidative stress and reduce NO bioavailability. This, in turn, diminishes PKG signaling, decreases titin phosphorylation, and increases passive cardiomyocyte stiffness [75].

Ordinarily, heart diseases do not only involve dysfunctions of a single organ. There are mechanisms throughout the body that interact with each other. For instance, during heart failure in mice, proinflammatory monocytes in the spleen are persistently mobilized to the heart. Therefore, splenectomy decreases tissue macrophage infiltration and halts adverse myocardial remodeling [91]. Likewise, macrophages play an important role in signaling processes during the interaction between the heart, brain, and kidneys, supporting adaptive mechanisms after cardiac pressure overload. Using wild-type mice subjected to transverse aortic constriction (TAC) and CD-*Klf5*KO mice (i.e., mice without kruppel-like factor 5 in renal collecting ducts). Fujiu et al. demonstrated that the sympathetic nervous system is activated in response to stress. Through a variety of mechanisms in the kidney and renal macrophage activity, renal endothelial cells secrete granulocyte–macrophage colony-stimulating factor (GM-CSF) into the blood circulation. GM-CSF induces the proliferation of cardiac Ly6C^lo^ macrophages, which produce amphiregulin (AREG), an important cardioprotective mediator. AREG modulates adaptive cardiac hypertrophy and fibrosis [92]. Moreover, neurohormonal pathways and their interaction with the immune system are also crucial in HF progression [93].

### Hypertension

Essential hypertension is characterized by chronic elevation of blood pressure and is associated with cardiovascular dysfunctions that may lead to HF. Many studies have been focused on the factors (both genetic and environmental) associated with the development of hypertension, e.g., associated with the function and regulation of the renin–angiotensin–aldosterone (RAA) system [94–96]. However, it is only in recent years that attention has been paid to immune cells, which also take part in the pathogenesis of hypertension [97–100]. Macrophages are mediators in the development of hypertension, which is associated with LV hypertrophy, cardiac remodeling, and fibrosis [101]. The level of macrophage infiltration in a hypertensive heart increases, and one of the mechanisms mediating this process is the CXCL1–CXCR2 axis [81, 102, 103].

Interestingly, one of macrophage functions is the regulation of blood pressure, extracellular fluid, and solute volume. This unique role was examined in rats treated with a high-salt diet, which caused interstitial hypertonic environment in the skin. In response to sodium accumulation the number of macrophages increases, simultaneously activating the tonicity-responsive enhancer binding protein (TonEBP) which senses the osmotic tissue pressure. In response to increased tissue tonicity, macrophages exhibiting the TonEBP receptor release vascular endothelial growth factor-C (VEGF-C), thus stimulating lymphangiogenesis. Therefore, the growth of lymphatic vessels during excessive salt intake blunts an increase of blood pressure, acting as a fluid buffering system. Moreover, the elevated serum levels of VEGF-C in humans with hypertension may indicate a similar mechanism in patients [104, 105]. Macrophage infiltration and upregulation of the TonEBP/VEGF-C signaling pathway also occur in the myocardium of spontaneously hypertensive rats under the influence of high-salt diet. Importantly, VEGF-C secretion and cardiac lymphangiogenesis lead to severe LV remodeling, which manifests by LV enlargement and perivascular and interstitial fibrosis [106].

### Obesity

Obesity is an independent risk factor of HF, as it causes hemodynamic changes that contribute to cardiac remodeling, which is manifested by changes in cardiac morphology and function [107]. Obesity correlates with worse myocardial symptoms in patients with HF [108] and greater systemic and local inflammation [109]. The level of monocytes increases in the circulation [110], and macrophages accumulate in the adipose tissue, especially in the epicardial adipose tissue (EAT) [111–113]. There are studies suggesting that macrophages can participate in the process of adaptive thermogenesis by the production of catecholamines [114]. However, this hypothesis has been recently challenged, showing that macrophages residing adjacent to neurons do not synthesize catecholamines [115] but are able to import and metabolize catecholamines, such as neuron-derived norepinephrine (NE) [116]. The NE transporter SLC6A2 and degradation enzyme MAOA are involved in these processes. A reduction in NE availability in the adipose tissue leads to blunted lipolysis, and thus promotes obesity [116]. Macrophages are known to be very susceptible to microenvironmental changes, particularly, to metabolic dysregulation, which is also mediated by obesity [117]. Many factors, such as hypoxia-induced signaling pathways [80, 118], metabolic substrates (e.g., fatty acids), proinflammatory lipid mediators [119–121], and hormones (e.g., leptin) [122], contribute to excessive macrophage activation. These factors stimulate a switch in the macrophage phenotype from anti-inflammatory to proinflammatory [123]. This leads to macrophages releasing proinflammatory cytokines, whose excess may contribute to microvascular abnormalities, fibrosis, and promote HF [80].

### Diabetes

The course of diabetes is related to a prolonged inflammatory response, which could induce adverse cardiac remodeling and increase the risk of HF [124]. Hyperglycemia stimulates macrophage infiltration, which produces inflammatory cytokines, such as TNF-α, IL-6, MCP-1, IL-1β. These cytokines, through various mechanisms, promote chronic inflammation, fibrosis, and tissue damage [125–127]. For instance, in diabetic mice macrophage-dependent, IL-1β production induced by toll-like receptor 2 (TLR2) and NLRP3 inflammasome activation may lead to cardiac arrhythmias [126]. IL-1β induction also reduces proangiogenic and regenerative capacity of myeloid angiogenic cells (MACs) under diabetic conditions, which may contribute to ischemic disease [128]. Diabetes results in the production of advanced glycation end-products (AGEs), which induce macrophage polarization towards an M1 phenotype via the RAGE/NF-κB pathway, and the secretion of TNF-α, IL-1β, IL-6 by macrophages [129]. Moreover, AGEs contribute to microvascular and macrovascular complications by increasing endothelial permeability and blocking nitric oxide (NO) activity in the endothelium, which generates reactive oxygen species (ROS). AGEs also induce cross-linking of collagen, thus promoting ventricular rigidity [130, 131]. Furthermore, in response to diabetes-related cardiac myocyte lipid overload, inflammatory pathways are activated, which often leads to lipotoxic cardiomyopathy. Macrophages are recruited to the heart even before the onset of cardiomyopathy, and the expression of their cytokines/cytokine receptors, e.g., IL-6, MCP-1, -2, -3, -5, CCR5, osteopontin, is upregulated. This leads to rapid progression of cardiac dysfunction. Depletion of macrophages reduces the expression of inflammatory markers and attenuates adverse disturbances in the heart [132].

One important aspect of diabetes is impaired macrophage phagocytic and chemotactic function, e.g., an insufficient efferocytosis of apoptotic cardiomyocytes and cellular debris [133]. In vitro studies indicated that under high glucose conditions, mir-126 expression in macrophages decreases, leading to upregulation of ADAM9 expression and MerTK cleavage. Apart from defective macrophage efferocytosis, loss of MerTK function results in a diminished capacity to resolve inflammation, which could contribute to defective organ repair. The same mechanism was also observed in human diabetic hearts [124]. These examples show how crucial is the role of macrophages in diabetes, potentially leading to heart dysfunction and damage of myocardial cells.

### Myocardial infarction

Acute MI is one of the main factors leading to HFrEF. In MI, macrophages play an extremely important role, which was widely studied [134]. Already in the first minutes after the injury, a cascade of reactions is initiated. These reactions include augmented adhesion molecule expression by endothelial cells, such as selectins and VCAM1, production of damage-associated molecular patterns (DAMPs) by dying cardiomyocytes, and the production of chemokines/cytokines, growth factors by resident macrophages, fibroblasts, and other cells [135]. This results in an influx of neutrophils, and monocytes from the bone marrow and spleen to the MI zone [136]. In the initial post-MI phase, the number of tissue-resident macrophages in the damaged region is significantly reduced, and they are replaced by proinflammatory macrophages [27, 28]. Cardiomyocyte death affects tissue-resident CCR2^+^ macrophages, which through MYD88-dependent mechanisms and increased myocardial CCL2/MCP-1 expression contribute to Ly6C^hi^ monocytes infiltrating the heart muscle and differentiating into proinflammatory Ly6C^hi^ macrophages [137, 138]. Moreover, resident CCR2^+^ monocyte-derived macrophages play an essential role in neutrophil extravasation by the production of CXCL2 and CXCL5 [139]. Within the first days after MI, the main macrophage function is the clearance of cell debris, especially dying cardiomyocytes [140]. Inflammatory cytokines, such as TNF-α, IL-1, IL-6, and MMPs, are also released, amplifying the inflammatory process [141]. The second phase, called the reparative phase, is dominated by Ly6C^low^ macrophages which could be differentiated from Ly6C^hi^ monocytes. The transcription factor Nr4a1 has been suggested to take part in switching from proinflammatory to anti-inflammatory mechanisms [137]. Ly6C^low^ macrophages are required for wound healing and reducing the inflammatory reaction. These macrophages start to release IL-10 and TGF-β, which promote collagen production, fibrosis, and angiogenesis [142]. Recent studies suggest that macrophages could directly contribute collagen to scar formation in zebrafish and mouse injured hearts [143]. Interestingly, the macrophages located in the pericardial cavity and exhibiting Gata6^+^ phenotype play a cardioprotective function after MI once they enter the myocardial wall. These macrophages are also involved in preventing fibrosis in a healthy myocardium [144]. Suppression of excessive fibrosis in the heart tissue is also mediated by the Ly6C^hi^ macrophages that accumulate in hypoxic areas and secrete Oncostatin-M, a member of IL-6 cytokine family, which inhibits fibroblast activation by downregulating αSMA expression [145].

Macrophages have a profound impact on the course of inflammation, the reparative phase, and preservation of cardiac functionality. However, when the balance between pro- and anti-inflammatory phases is not maintained, for instance when the inflammatory reaction is prolonged or inadequately suppressed, it could lead to serious pathological consequences, including impaired systolic function and adverse LV remodeling [142].

### Heart valve pathology/remodeling

Valvular heart dysfunction is a disease whose prevalence increases with age, with more than 10% of adults older than 75 years affected. Valvular dysfunction is frequently associated with mitral valve fibrosis and remodeling, as observed as a consequence of an autoimmune response in rheumatic heart disease [146, 147]. In a mouse model of mitral valve disease CD301b^+^, mononuclear phagocytes accumulate in the valve. These phagocytes are involved in the processes of inflammation and fibrosis within the valve and are characterized by the presence of other markers: CD11b, CD64, CX3CR1, and expression of tissue-reparative Arg-1 and FIZZ-1 and the proinflammatory cytokines IL-6 and TNF-α. These processes are accompanied by a local proliferation of myeloid cells and a recruitment of circulating cells. The latter event takes place through VCAM-1 and very late antigen-4 (VLA-4) upregulation. Likewise, studies on samples of cardiac valve tissue with human rheumatic myocarditis confirmed the presence of CD301b^+^ cells in the valves [148].

Myxomatous valve disease is another valve pathology in which macrophages are involved. This disease is associated with valve leaflet thickening and extracellular matrix disruption. In the regions of aortic and mitral valves where modified heavy chain hyaluronan is deposited, there is an increased number of macrophages compared with that in the valves of healthy individuals. Proinflammatory CCR2^+^ monocytes are recruited, which is likely promoted by damaged endothelial cells, increased expression of chemokines and interleukins, and hyaluronan accumulation. On the other hand, the population of CD206^+^ macrophages also increases. These macrophages play a role in promoting tissue repair and regeneration [45].

## Conclusions and future aspects

This article aims to present the latest reports on the origin, phenotypes, and functions of cardiac tissue macrophages in healthy hearts and in cardiovascular diseases. Macrophages are necessary for proper heart development and functioning. However, in injured hearts, these cells can play beneficial and/or detrimental roles; this is often related to their phenotype, origin, accumulation in local tissue, and easy modulation by adjacent microenvironmental changes. Due to the fact that current treatments are insufficient in blocking the development and progression of heart failure, cardiac macrophages may constitute a potential therapeutic target. For instance, the population of proinflammatory CCR2^+^ macrophages has been suggested as a potential novel target in heart failure treatment [41]. Using CCR2-targeted siRNAs, encapsulated in a lipid nanoparticle, monocyte infiltration was reduced, which also attenuated infarct inflammation, and LV remodeling [149]. Interestingly, after ischemia/reperfusion, cardiac stem-cell therapy induces the temporal and regional accumulation of CCR2^+^ and CX3CR1^+^ macrophages, which rejuvenate the infarcted area and improve cardiac function [42]. Furthermore, stromal cell-derived exosomes, which modify macrophages towards a cardioprotective phenotype, are a promising tool for further therapeutic trials [150]. Therapies related to introducing modifications at the miRNA level are worth considering as well. One of mechanism which underlies cardioprotective effect is associated to exosomal transfer of miR-181b from cardiosphere-derived cells into macrophages, in a rat model of MI [151]. Moreover, post-MI nanoparticle delivery of miR-21 to cardiac macrophages was shown to switch the macrophage phenotype from pro-inflammatory to anti-inflammatory, promote angiogenesis, and reduce hypertrophy, fibrosis, diminishing an adverse myocardial remodeling [152]. Inhibition of monocyte recruitment to the heart after injury may preserve the embryonic macrophage population in the myocardium, reduce inflammation, and enhance tissue repair [24]. Treatment with Enalapril (ACE inhibitor) reduced splenic release of monocytes and their infiltration into the healing infarct, which in consequence improved the ejection fraction by 14% [153]. Moreover, targeting inflammatory cytokines and chemokines, such as IL-1, IL-6, CCL2, TGF-β, TNF-α, could be helpful in heart failure treatment [154, 155]. For instance, suppression of chemokine receptor CXCR7 inhibits M1 macrophage polarization, chemotaxis, and inflammation to improve post-MI injury [156]. Therefore, macrophages have a therapeutic potential, and a thorough understanding of their role in the progression of heart failure is crucial.
